# Diagnostic Accuracy of Interferon-Gamma Release Assays for Tuberculous Meningitis: A Systematic Review and Meta-Analysis

**DOI:** 10.3389/fcimb.2022.788692

**Published:** 2022-04-22

**Authors:** An Wen, Er-Ling Leng, Shi-Min Liu, Yong-Liang Zhou, Wen-Feng Cao, Dong-Yuan Yao, Fan Hu

**Affiliations:** ^1^ Department of Neurology, Jiangxi Provincial People’s Hospital (The First Affiliated Hospital of Nanchang Medical College), Nanchang, China; ^2^ Institution of Neurology, Jiangxi Provincial People’s Hospital (The First Affiliated Hospital of Nanchang Medical College), Nanchang, China; ^3^ Department of Pediatrics, Jiangxi Provincial People’s Hospital (The First Affiliated Hospital of Nanchang Medical College), Nanchang, China

**Keywords:** tuberculous meningitis, cerebrospinal fluid, meta-analysis, interferon-release assays, tuberculosis

## Abstract

**Background:**

In this study, we evaluated and compared the accuracy of blood and cerebrospinal fluid (CSF) interferon release tests [interferon-gamma release assays (IGRAs)] in the diagnosis of tuberculous meningitis (TBM) by a meta-analysis of the relevant literature.

**Methods:**

We searched for studies published before 2021 in Medline, Embase, the Cochrane database, and Chinese databases. All studies used the QuantiFERON-TB Gold In-Tube and/or T-SPOT.TB method. Blood and/or CSF tests that met the guidelines for the quality assessment of studies with diagnostic accuracy were included. We used the revised diagnostic accuracy study quality assessment to assess the quality of the included studies. Begg’s funnel plots were used to assess publication bias in the meta-analysis of the diagnostic studies, and statistical analyses were performed by using Stata (Version 12) software.

**Results:**

A total of 12 blood and/or CSF IGRA studies were included in this meta-analysis, with 376 patients and 493 controls. The sensitivity, specificity, positive likelihood ratio, negative likelihood ratio, diagnostic odds ratio, and area under the summary receiver operating characteristic curve (SROC) of the blood IGRAs in the pooled data from 12 studies were 74% (95% CI: 0.65-0.82), 78% (95% CI: 0.68-0.86), 3.38 (95% CI 2.26-5.06), 0.33 (95% CI: 0.23-0.46), 10.25 (95% CI: 5.46-19.25), and 0.83 (95% CI: 0.79-0.86), respectively. For CSF IGRAs, these values for the pooled data from the 10 studies included were 79% (95% CI: 0.71-0.85), 95% (95% CI: 0.88-0.98), 16.30 (95% CI 6.5-40.83), 0.22 (95% CI: 0.16-0.31), 57.93 (95% CI: 22.56-148.78), and 0.91 (95% CI: 0.88-0.93), respectively.

**Conclusion:**

CSF IGRAs exhibited a better diagnostic accuracy than blood IGRAs in diagnosing TBM.

## Introduction

The World Health Organization (WHO) Global Tuberculosis Report 2020 estimates the number of people living with tuberculosis (TB) at around 10 million in 2019, which makes it the most common cause of death due to a single infectious agent (Chakaya et al., 2021). Tuberculous meningitis (TBM), the most serious form of extrapulmonary TB (Brancusi et al., 2012), is caused by *Mycobacterium tuberculosis* (*M. tuberculosis*, MTB) and is associated with significant morbidity and mortality, especially among children and people living with HIV (Ho et al., 2013). Early diagnosis and treatment of TBM is crucial for its prognosis (Garg, 2010). Unfortunately, early diagnosis of TBM is often difficult. Cerebrospinal fluid (CSF) smear, *M. tuberculosis* culture, and polymerase chain reaction are the gold standards for detecting *M. tuberculosis* in the CSF (Marais et al., 2010). However, the possibility of identifying acid-fast bacilli in CSF smears is very low, and the culture of *M. tuberculosis* in CSF is time consuming (Thwaites et al., 2004; Garg, 2019). Although polymerase chain reaction has a higher sensitivity in detecting *M. tuberculosis* DNA in CSF samples, it also has a higher false-positive rate (Donovan et al., 2020). In response to these challenges, a new technique for the rapid detection of *M. tuberculosis* has been developed.

In the past decade, the interferon-gamma release assay (IGRA) has become widely used as an immunodiagnostic method for *M. tuberculosis* infection (Bergot et al., 2018). It detects interferon (IFN)-gamma produced by T cells as a reaction to *M. tuberculosis*-specific antigens, such as early secretory antigenic target (ESAT)-6 and culture filtrate protein (CFP)-10, which are thought to be present only in *M. tuberculosis* but not in *M. bovis* bacille Calmette–Guérin (BCG) vaccine and other mycobacteria (Pai et al., 2006). QuantiFERON-TB Gold In-Tube (QFT-G-IT) (Cellestis, Carnegie, VIC, Australia) and T-SPOT.TB (T-SPOT) (Oxford Immunotec, Abingdon, United Kingdom) are the two most widely used IGRA systems to date, using enzyme-linked immunosorbent assay (ELISA) and enzyme-linked immunospot assay (ELISPOT) to detect IFN-gamma (Pai et al., 2006).

Blood IGRAs are most commonly used. However, an alternative approach of IGRAs using effector T cells from infected TB site specimens may be more likely to detect IFN against TB infection than using peripheral blood mononuclear cells (PBMCs). Recent studies have evaluated the use of CSF IGRAs for the diagnosis of TBM; however, the sample sizes of these included studies were insufficient, and their accuracy was disputable. Therefore, this study aimed to perform a meta-analysis to systematically evaluate and compare the accuracy of blood and CSF IGRAs in the diagnosis of TBM and to review relevant literature.

## Methods and Materials

### Search Strategy

Relevant works of literature in English were retrieved using Web of Science, PubMed, EBSCO, Medline, Elsevier, and Cochrane Library, while those in Chinese were retrieved using Wanfang Data, China Biology Medicine discs, and China Knowledge Resource Integrated Database. The following keywords were used as search terms: “Tuberculosis meningitis”, “Mycobacterium tuberculosis”, “Tuberculosis”, “Interferon-gamma release assay”, “T cell-based assay”, “T-SPOT.TB”, “ELISPOT”,”IGRA”, “Quantiferen”, “ESAT-6”, “CFP-10”, “Cerebrospinal fluid”, “Sensitivity”, “Specificity”, and “Accuracy”. The study included all diagnostic studies published before 2021.

### Study Selection

The inclusion criteria were as follows: (1) IGRAs including T-SPOT (ELISPOT) and/or QFT-GIT were used for the diagnosis of TBM; (2) blood and/or CSF IGRAs were performed; and (3) research articles with original data were included. The exclusion criteria include the following: (1) duplicated studies, case reports, reviews, animal studies, and abstracts; (2) studies without any control group; (3) IGRA systems performed other than QFT-GIT and T-SPOT (ELISPOT); and (4) the fourfold table was not presented or could not be provided.

### Data Extraction

Two reviewers independently extracted the data using standard data extraction forms (**Table 1**). The data extracted from these selected articles were as follows: study sites, the date of publication, author’s name, population studied, assay type, diagnostic method of TB, and cut-off of IGRAs. Inconclusive results have been eliminated. Discrepancies were resolved by a third examiner.

**Table 1:** Characteristics of the included studies.

	Country	Sample size(n/N)	Age(years)	Design	QUADUS score	Blingding	Type of IGRA	Cut off
Kim et al., 2008	Korea	12/25	45.5 ± 16.5/39.3 ± 16.5^‡^	Prospective	13	Yes	ELISPOT	CSF/Blood ≥2
Thomas et al., 2008	India	11/9	32(8-69)/36(16-64)^§^	Prospective	14	Yes	ELISPOT	Manufacturers’ instructions (Blood~CSF)
Kim et al., 2010	Korea	31/55	45.4 ± 14.8/44.0 ± 19.6^‡^	Prospective	12	Yes	ELISPOT	≥6SFC(Blood); ≥6SFC (CSF)
Patel et al., 2010	South Africa	38/48	33.5 ± 9.5/32.9 ± 9.7^‡^	Prospective	11	Unclear	T-Spot.TB	≥46SFC(Blood); ≥46SFC (CSF)
Vidhate et al., 2011	India	36/16	28.9 ± 11.8/34.5 ± 17.1^‡^	Unknown	13	Unclear	QFT-GIT	>0.35 IU/ml (Blood)
Cho et al., 2011	Korea	35/87	48.3 ± 16.1/48.1 ± 17.6^‡^	Prospective	10	Yes	T-Spot.TB	≥6SFC (Blood)
Park et al., 2012	Korea	25/57	≥16^Ɨ^	Prospective	6	Unclear	T-Spot.TB	≥6SFC (Blood); ≥6SFC (CSF)
Zhang et al., 2013	China	30/30	17-74^Ɨ^	Unknown	10	Unclear	T-Spot.TB	Manufacturers’ instructions (Blood~CSF)
Qin et al., 2015	China	12/28	46 (24-59)/43(29-55)^¶^	Unknown	13	Yes	T-Spot.TB	≥6SFC(Blood); ≥6SFC(CSF)
Caliman-Sturdza et al., 2015	Romania	63/62	0.7-17.2/1.2-17.2^Ɨ^	Unknown	13	Unclear	QFT-GIT	>0.35IU/ml (Blood); >0.35 IU/ml (CSF)
Lu et al., 2016	China	30/39	45 (18-79)/36(14-64)§	Unknown	11	Unclear	QFT-G-IT/ELISPOT	Manufacturers’ instructions (Blood~CSF)
Pan et al., 2017	China	53/37	31(18-79)/36(14-47)^¶^	Prospective	12	Yes	ELISPOT	Manufacturers’ instructions (Blood); ≥24SFC (CSF)

IGRA, interferon-gamma release assay; CSF, cerebrospinal fluid; AFB, acid-fast bacilli; PCR, polymerase chain reaction; QFT-G-IT, QuantiFERON-TB Gold in-tube; ELISPOT, enzyme-linked immunospot; SFC, spot-forming cell.

^Ɨ^Mean.

^‡^Mean ± SD.

^§^Mean (range).

^¶^Median (IQR).

### Quality Assessment

The quality of the studies that aimed to calculate the accuracy of the analyses was assessed independently by two reviewers using the Quality Assessment of Diagnostic Accuracy Studies (QUADAS-2) checklist, in which each assessment item was assigned the labels “yes”, “no”, or “unclear” (Whiting et al., 2011).

### Statistical Analysis

The degree of heterogeneity of the selected studies was evaluated using the *Q*-test, and the size of the heterogeneity was quantified by computing for *I* ^2^. For each study, 2 × 2 tables representing true-positive, true-negative, false-positive, and false-negative values were identified. The sensitivity, specificity, diagnostic odds ratio (DOR), positive likelihood ratio (PLR), negative likelihood ratio (NLR), and area under the summary receiver operating characteristic (SROC) curve from the pooled data were calculated by meta-analysis and expressed in 95% confidence intervals (CIs). Begg’s funnel plot was used to assess publication bias in nine of the included studies. Fagan’s nomogram was used to calculate the post-test probability for each group. All data were analyzed using STATA version 12.0 software. Statistical significance was set at *P <*0.05.

## Results

### Characteristics of the Included Studies

**Figure 1:**
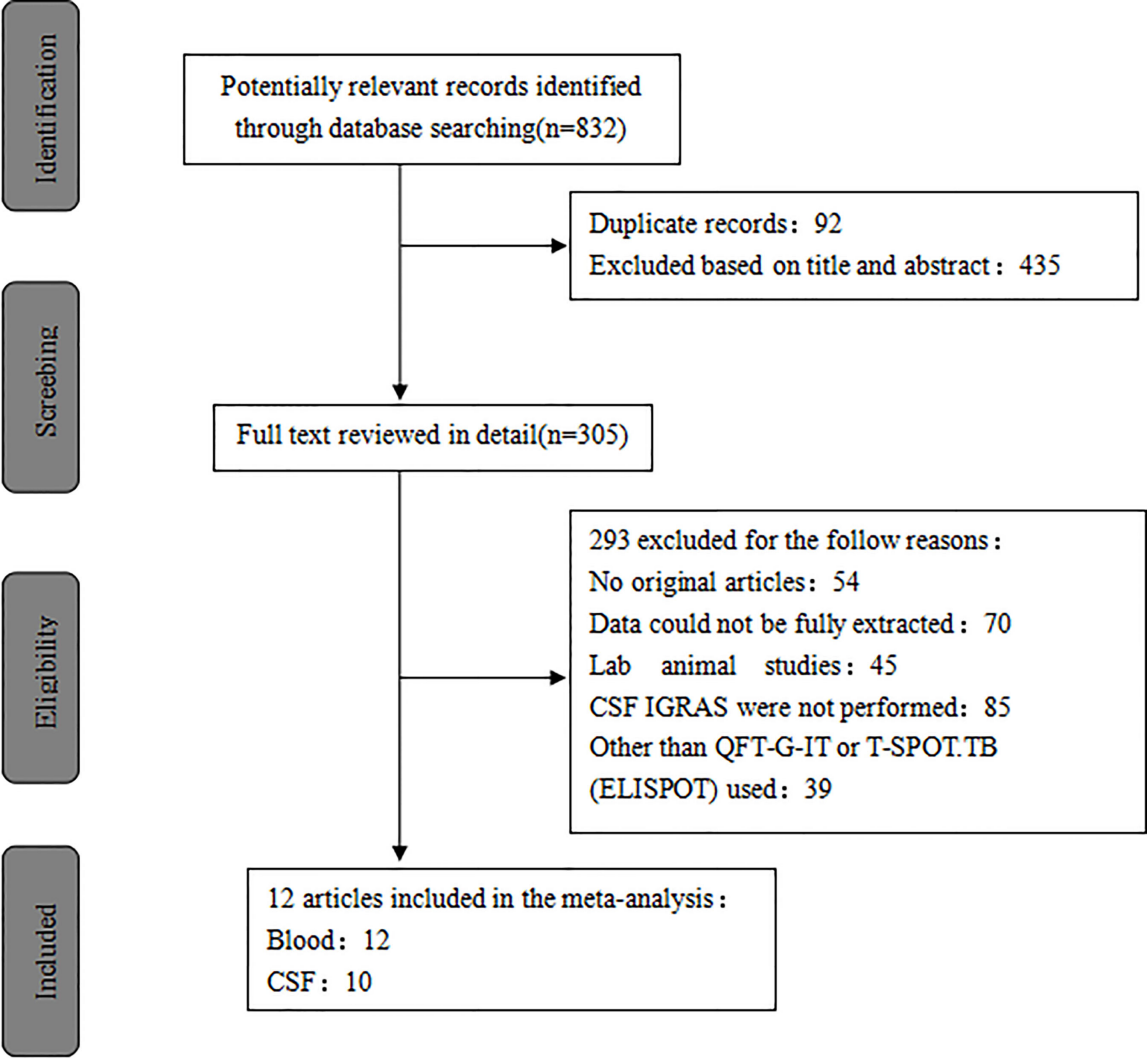
The study selection process flowchart.

A total of 832 published studies were screened using the process shown in **Figure 1**. Among these, 12 studies with 854 human subjects (386 TB patients and 468 controls) from five countries satisfied the inclusion criteria (Kim et al., 2008; Thomas et al., 2008; Kim et al., 2010; Patel et al., 2010; Cho et al., 2011; Vidhate et al., 2011; Park et al., 2012; Zhang et al., 2013; Caliman-Sturdza et al., 2015; Qin et al., 2015; Lu et al., 2016; Pan et al., 2017) including six prospective case-controlled studies (Kim et al., 2008; Thomas et al., 2008; Kim et al., 2010; Patel et al., 2010; Cho et al., 2011; Park et al., 2012; Pan et al., 2017). Most of the studies included were from Asia, of which four were from South Korea (Kim et al., 2008; Kim et al., 2010; Cho et al., 2011; Park et al., 2012), four from China (Zhang et al., 2013; Qin et al., 2015; Lu et al., 2016; Pan et al., 2017), and two from India (Kim et al., 2008; Vidhate et al., 2011). The remaining studies were conducted either in South Africa (Patel et al., 2010) or Romania (Caliman-Sturdza et al., 2015). Blood IGRAs were performed in all, CSF IGRAs (Kim et al., 2008; Thomas et al., 2008; Kim et al., 2010; Patel et al., 2010; Park et al., 2012; Zhang et al., 2013; Caliman-Sturdza et al., 2015; Qin et al., 2015; Lu et al., 2016; Pan et al., 2017) in ten, IGRAs using the T-Spot or ELISPOT system (Kim et al., 2008; Thomas et al., 2008; Kim et al., 2010; Patel et al., 2010; Cho et al., 2011; Park et al., 2012; Zhang et al., 2013; Qin et al., 2015; Pan et al., 2017) in nine, and the QFT-G-IT system (Vidhate et al., 2011; Caliman-Sturdza et al., 2015; Lu et al., 2016) in three of the 12 included studies (**Table 1**). Eleven studies (Kim et al., 2008; Thomas et al., 2008; Kim et al., 2010; Patel et al., 2010; Cho et al., 2011; Vidhate et al., 2011; Zhang et al., 2013; Caliman-Sturdza et al., 2015; Qin et al., 2015; Lu et al., 2016; Pan et al., 2017) were performed on patients with culture-confirmed TB. In this study, testing of 60 case individuals yielded a positive result for TBM *via* CSF culture and/or AFB, with a sensitivity of only 16% (**Table 2**).

**Table 2:** Principal data characteristics of included studies.

	Year	Country	TBM patients	Diagnostic methods(N)	IGRA methods	Sample	Test result			
							TP	FP	FN	TN
Kim	2008	Korea	12	Culture(4),PCR(3),AFB(1)	ELISPOT	PB	10	9	1	15
						CSF	3	3	1	9
Thomas	2008	India	11	Culture(1),PCR(1)	ELISPOT	PB	9	2	2	6
						CSF	9	0	1	7
Kim	2010	Korea	31	Culture(7),PCR(4),AFB(4)	ELISPOT	PB	22	9	20	30
						CSF	13	1	5	25
Patel	2010	South Africa	38	Culture,PCR:(Unclear)	T-Spot.TB	PB	22	3	16	45
						CSF	31	0	7	48
Vidhate	2011	India	36	Culture(1),PCR(13),AFB(2)	QFT-GIT	PB	16	6	20	10
Cho	2011	Korea	35	Culture,PCR:(Unclear)	T-Spot.TB	PB	26	47	9	40
Park	2012	Korea	25	unclear	T-Spot.TB	PB	22	24	3	33
						CSF	18	12	7	45
Zhang	2013	China	30	Culture,PCR:(Unclear)	T-Spot.TB	PB	23	4	7	26
						CSF	28	1	2	29
Qin	2015	China	12	Culture(2),PCR(1),AFB(1)	T-Spot.TB	PB	10	2	5	23
						CSF	11	1	2	26
Caliman-Sturdza	2015	Romania	63	Culture,AFB:(positive 25)	QFT-GIT	PB	49	7	13	51
						CSF	45	1	11	55
Lu	2016	China	30	Culture,AFB:(positive 6)	QFT-G-IT/ELISPOT	PB	21	5	7	34
						CSF	25	6	5	33
Pan	2017	China	53	Culture(5),PCR(15),pathology(1)	ELISPOT	PB	48	9	5	28
						CSF	32	1	21	36

IGRA, interferon-gamma release assay; PB, peripheral blood; CSF, cerebrospinal fluid; AFB, acid-fast bacilli; PCR, polymerase chain reaction; QFT-G-IT, QuantiFERON-TB Gold in-tube; ELISPOT, enzyme-linked immunospot; TP, true positive; FP, false positive; FN, false negative; TN, true negative.

### Quality of the Included Studies

This meta-analysis assessed the quality of the included studies using the QUADAS-2 tool, which contains 14 items in the checklist. Among the 12 included studies, one (Thomas et al., 2008) met 14 items, four (Kim et al., 2008; Vidhate et al., 2011; Caliman-Sturdza et al., 2015; Qin et al., 2015) met 13 items, two (Kim et al., 2010; Pan et al., 2017) met 12 items, another two (Patel et al., 2010; Lu et al., 2016) met 11 items, two more (Cho et al., 2011; Zhang et al., 2013) met 10 items, and the last one (Park et al., 2012) met 6 items in the checklist.

### Diagnostic Accuracy of Interferon-Gamma Release Assays in Blood

**Figure 2:**
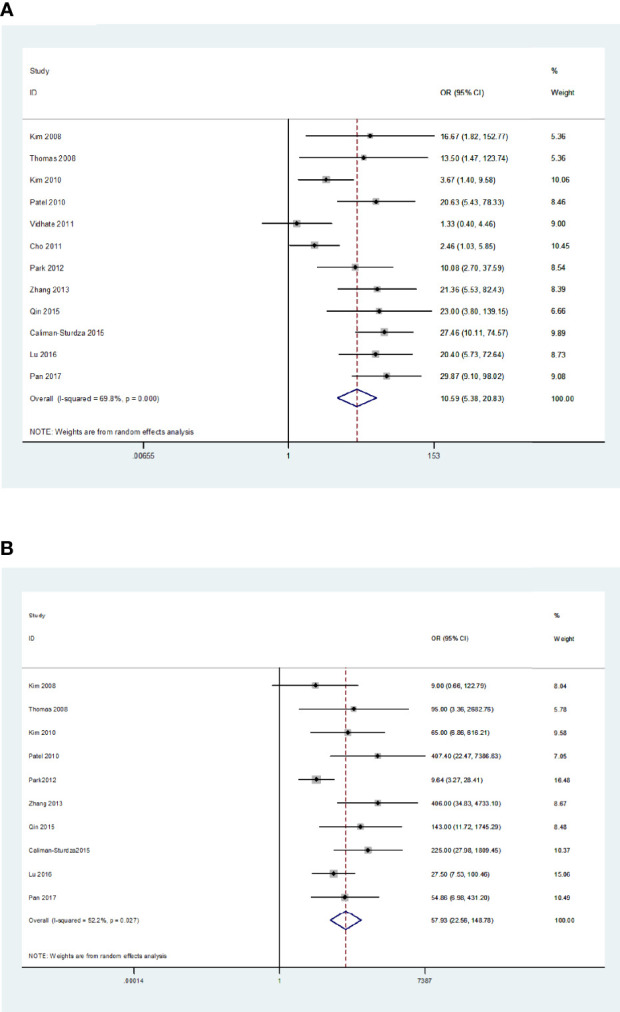
Diagnostic accuracy of the IGRA. For blood IGRAs **(A)**, pooled sensitivity and specificity were 74% (95% CI: 0.65-0.82) and 78% (95% CI: 0.68-0.86), respectively. Moreover, PLR, NLR, and DOR were 3.38 (95% CI: 2.26-5.06), 0.33 (95% CI: 0.23-0.46), and 10.25 (95% CI: 5.46-19.25). For CSF IGRAs **(B)**, pooled sensitivity and specificity were 79% (95% CI: 0.71-0.85) and 95% (95% CI: 0.88-0.98), respectively. In addition, PLR, NLR, and DOR were 16.30 (95% CI: 6.50-40.83), 0.22 (95% CI: 0.16-0.31), and 57.93 (95% CI: 22.56-148.78), respectively.

**Figure 3:**
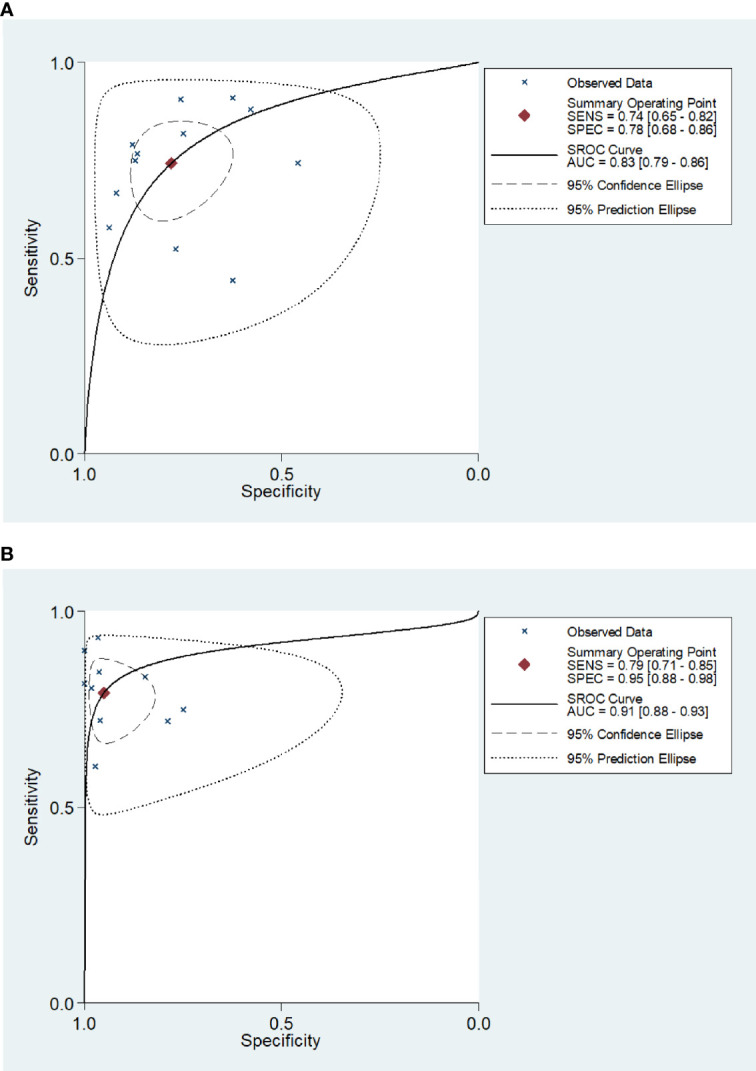
Receiver operating characteristic curve of the IGRA for diagnosis of tuberculosis meningitis. The SROC curves for blood **(A)** and CSF IGRAs **(B)**. The AUCs were 0.83 (95% CI: 0.79-0.86) for blood IGRAs **(A)** and 0.91 (95% CI: 0.88-0.93) for CSF IGRAs **(B)**.

The *Q*-test and *I^2^* statistic results showed high heterogeneity among the included studies (*P*=0.000, *I^2^* >50%). As a result, a random-effects model was used for the meta-analysis. The sensitivity and specificity of blood IGRAs obtained from pooled data were 74% (95% CI: 0.65-0.82) and 78% (95% CI: 0.68-0.86), respectively. In addition, the PLR, NLR, and DOR were 3.38 (95% CI: 2.26-5.06), 0.33 (95% CI: 0.23-0.46), and 10.25 (95% CI: 5.46-19.25), respectively. The false-positive rate of blood detected by T-SPOT was 0.307 and the false-negative rate was 0.298, while QFT-GIT is 0.159 and 0.317, respectively. The area under the SROC curve of blood IGRA was 0.83 (95% CI: 0.79-0.86) (**Figures 2A** and **3A**).

### Diagnostic Accuracy of Interferon-Gamma Release Assays in Cerebrospinal Fluid

The *Q*-test and *I^2^* statistic results also showed high heterogeneity among the included studies that utilized CSF as the medium analyzed (*P*=0.03, *I^2^* >50%). Therefore, a random-effects model was used for the meta-analysis. The sensitivity and specificity of CSF IGRAs obtained from pooled data were 79% (95% CI: 0.71-0.85) and 95% (95% CI: 0.88-0.98), respectively. In addition, the PLR, NLR, and DOR were 16.30 (95% CI: 6.50-40.83), 0.22 (95% CI: 0.16-0.31), and 57.93 (95% CI: 22.56-148.78), respectively. The false-positive rate of CSF detected by T-SPOT was 0.078 and the false-negative rate was 0.24, while QFT-GIT is 0.074 and 0.21, respectively. The AUC of the SROC curve was 0.91 (95% CI: 0.88-0.93) for CSF IGRAs (**Figures 2B** and **3B**). Therefore, CSF IGRAs showed higher diagnostic sensitivity and specificity than blood IGRAs.

### Publication Bias Analysis

**Figure 4:**
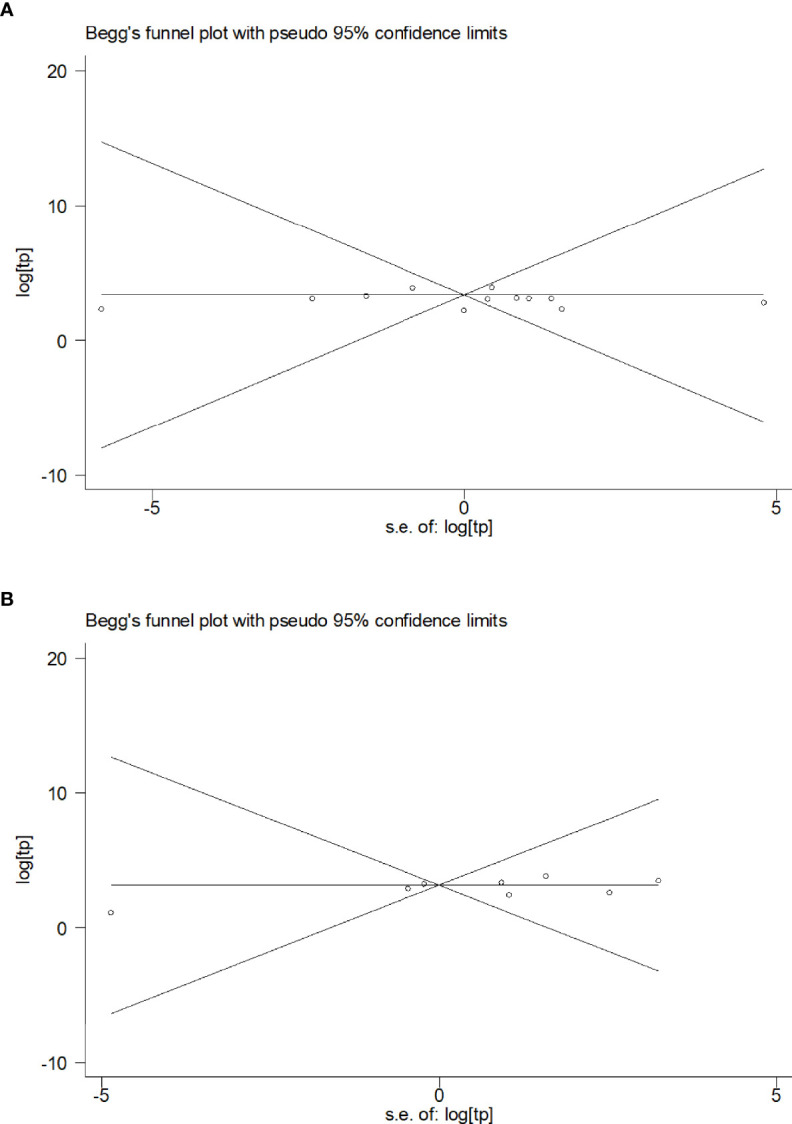
Funnel plot of the included studies. **(A)** Funnel plots of the included studies on blood **(A)** and CSF samples **(B)**.

The publication bias of the included studies was determined using Begg’s funnel plot. The Pr>|z| values of blood and CSF were 0.815 (**Figure 4A**) and 0.458 (**Figure 4B**), respectively, indicating that no publication bias was observed in blood or CSF IGRAs.

### Post-Test Probability of the Disease

**Figure 5:**
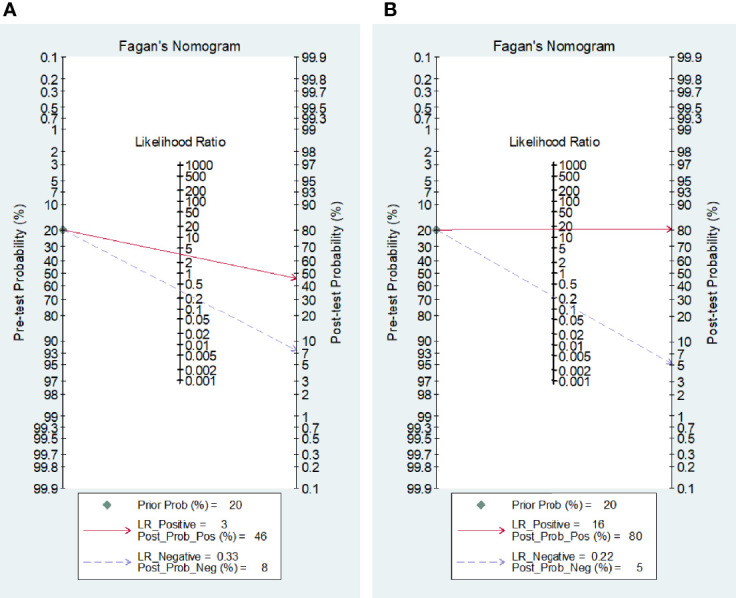
Post-test probabilities of tuberculosis for blood **(A)** and CSF IGRA **(B)**.

Fagan’s nomogram statistics showed that the positive post-test probabilities of TBM after either blood or CSF IGRAs were 46% and 80%, respectively (**Figures 5A, B**). A higher but limited probability of body fluids indicates that a positive IGRA result should not be used solely for the diagnosis of TBM, either from blood or from CSF. On the contrary, the negative post-test probabilities were 8% for blood and 5% for CSF IGRAs (**Figures 5A, B**), which indicates that negative body fluid IGRA results would be more reliable in excluding suspected TBM.

## Discussion

Since early diagnosis and treatment of TBM save lives, tests must be performed to diagnose TBM quickly and accurately, especially in its early stages (Ho et al., 2013). Unfortunately, the diagnosis of TBM is often ambiguous because of nonspecific clinical manifestations and the low sensitivity of available diagnostic methods (Thwaites et al., 2013). The absolute and most widely used diagnostic tools for TBM are Ziehl-Neelsen staining and culture (Marais et al., 2010), but they are negative in the majority of TBM cases. Our results showed that the sensitivity for CSF culture and/or AFB was 16% in the TBM group. Increasing the volume of CSF (>6 ml) obtained and meticulous microscopy (for at least half an hour) further increases the chance of positive diagnosis (Heemskerk et al., 2018; Bahr et al., 2019). For this reason, we did not restrict diagnostic criteria to microbiological confirmation, which is usually not possible in a routine clinical setting. This may produce bias. The detection of *M. tuberculosis* DNA in CSF samples using nucleic acid amplification tests (NAATs) are a widely used diagnostic method (Marais et al., 2010). In recent years, some studies on NAATs (Modi et al., 2016 Jyothy et al., 2019; Kwizera et al., 2019; Pormohammad et al., 2019; Siddiqi et al., 2019; Poplin et al., 2020; Slane and Unakal, 2021) reported a potential to rule in or confirm diagnosis (specificity, 80%-100%), but low sensitivity (~40%-96%) precludes the use of these tests to rule out TBM. Up to now, adenosine deaminase (ADA) is still a hot spot in TB diagnosis. Numerous studies have been published regarding the usefulness in TBM. Performance varies according to the assay and cut-off used, the mean sensitivity and specificity of ADA assays were 60%-90% and 80%-90%, respectively (Pormohammad et al., 2017). ADA assays may be useful in identifying TBM, but raised levels may also be seen in other central nervous system diseases such as purulent meningitis (Ekermans et al., 2017) (**Table 3**).

**Table 3:** Comparison of conventional and novel diagnostic tests for tuberculous meningitis performed on CSF specimens.

Diagnostic test in CSF specimens	Sensitivity (%)	Specificity (%)	Comments	References
Microbiological diagnosis				
Ziehl-Neelsen	10-40	100	Sensitivity substantially improved by meticulous microscopy of large volumes of CSF(>6 ml).	Bahr et al. (2019); Heemskerk et al. (2018)
Mycobacterial culture	50-60	100	Takes at least 2 weeks (and, in many cases, up to 6 weeks): clinicians cannot afford to wait for culture results before treating patients.	Bahr et al. (2019);
Nucleic acid amplification tests (NAATs)				
Xpert MTB/RIF	50-70	95-100	Good “rule in” test, but it does not appear to be adequate to rule out TBM. The requirements of trained laboratory staff and high costs. Requires further evaluation.	Jyothy et al. (2019); Slane and Unakal (2021)
LAMP	88-96	80-100		Modi et al. (2016); Siddiqi et al. (2019); Kwizera et al. (2019)
Amplicor TB PCR test	~40	90-100		Poplin et al. (2020)
MTD	86	99		Pormohammad et al. (2019)
Immune response-based diagnosis				
ADA	60-90	80-90	Variable results, cannot differentiate purulent meningitis from TBM.	Pormohammad et al. (2017); Ekermans et al. (2017)
CSF IGRAs	79% (95% CI: 0.71-0.85)	95% (95% CI: 0.88-0.98)	CSF IGRA is better at distinguishing ATB and has a higher ability to predict the location of *M. tuberculosis* infection, especially in TBM cases. Very few studies, small subject numbers. Furthermore, cut-off and incubation cell numbers across the studies were inconsistent.	Present

IGRA, interferon-gamma release assay; CSF, cerebrospinal fluid; LAMP, loop-mediated isothermal amplification; MTD, The Gen-probe amplified M. tuberculosis direct test; ADA, adenosine deaminase; ATB, active tuberculosis.

To address this, newer tests have emerged for the early diagnosis of TBM, such as IGRAs. The design and development of IGRAs were based on the fact that T lymphocytes release IFN-gamma in response to the stimulation of *M. tuberculosis*-specific antigens such as ESAT-6 and CFP-10 (Andersen et al., 2000; Pai et al., 2007; Stevens et al., 2019). Moreover, blood-based tests have been widely evaluated and are considered promising tools for the rapid detection of *M. tuberculosis* (Westermann and Pabst, 1992; Meier et al., 2005; Kawamura et al., 2012; Rangaka et al., 2012). In addition, the accuracy of CSF IGRAs has also been evaluated for the diagnosis of TBM (Kim et al., 2008; Thomas et al., 2008; Kim et al., 2010; Patel et al., 2010; Park et al., 2012; Zhang et al., 2013; Caliman-Sturdza et al., 2015; Qin et al., 2015; Lu et al., 2016; Pan et al., 2017). However, no studies comparing blood and CSF IGRAs have been performed yet. Therefore, we performed this meta-analysis to systematically evaluate and compare the diagnostic accuracy of blood and CSF IGRAs for diagnosing TBM. The obtained overall sensitivity, specificity, PLR, NLR, DOR, and the SROC AUC in blood samples were 74% (95% CI: 0.65-0.82), 78% (95% CI: 0.68-0.86), 3.38 (95% CI: 2.26-5.06), 0.33 (95% CI: 0.23-0.46), 10.25 (95% CI: 5.46-19.25), and 0.83 (95% CI: 0.79-0.86), respectively, and for CSF, these values were 79% (95% CI: 0.71-0.85), 95% (95% CI: 0.88-0.98), 16.30 (95% CI: 6.50-40.83), 0.22 (95% CI: 0.16-0.31), 57.93 (95% CI: 22.56-148.78), and 0.91 (95% CI: 0.88-0.93), respectively. The post-test probability of blood samples was 46%/8% and 80%/5% in CSF samples. These results suggest that the IGRAs carried out in CSF have better diagnostic sensitivity and specificity than IGRAs in blood for the diagnosis of TBM.

However, even if the sensitivity, specificity, and post-test probability were higher for CSF IGRAs than for blood IGRAs in this analysis, the diagnostic sensitivity of CSF IGRAs alone was not high enough to support the diagnosis of TBM. Nevertheless, CSF IGRAs have some advantages over blood IGRAs. When peripheral blood IGRAs are used alone, it becomes difficult to distinguish active tuberculosis (ATB) from latent tuberculosis infection in a clinical setting (Pai et al., 2004; Gao et al., 2017). In an ATB stimulation, antigen-specific T lymphocytes are recruited to the sites of infection and proliferate rapidly (Schwander et al., 1998; Hirsch et al., 2001). Because of this, CSF IGRA is better at distinguishing ATB and has a higher ability to predict the location of the *M. tuberculosis* infection, especially in TBM cases.

The current analysis had some limitations. First, among the ten included studies that utilized CSF IGRA, the sample size was fairly small. Second, the cut-off and incubation cell numbers across the studies were inconsistent. This is an important consideration because the cut-off values greatly influence the sensitivity and specificity. Finally, in some of the included studies, the diagnosis of TBM patients was not confirmed by microbiological methods (smear or culture).

## Conclusion

The results of this meta-analysis showed that CSF IGRAs had higher sensitivity and specificity in the diagnosis of TBM than peripheral blood IGRAs. However, carefully designed higher-quality independent studies are required to reliably compare the diagnostic accuracies of blood and CSF IGRAs.

## Author Contributions

AW, E-LL, and FH designed the study. The manuscript was written by AW, E-LL, and FH with the final approval. S-ML and Y-LZ did the data searches and study selection. W-FC and D-YY did the data synthesis and created the tables and figures. All authors contributed to the article and approved the submitted version.

## Funding

This study was supported by Jiangxi Provincial Health Commission Science and Technology Foundation grants 20203025.

## Conflict of Interest

The authors declare that the research was conducted in the absence of any commercial or financial relationships that could be construed as a potential conflict of interest.

## Publisher’s Note

All claims expressed in this article are solely those of the authors and do not necessarily represent those of their affiliated organizations, or those of the publisher, the editors and the reviewers. Any product that may be evaluated in this article, or claim that may be made by its manufacturer, is not guaranteed or endorsed by the publisher.
